# Psychotraumatology in Europe: a personal history

**DOI:** 10.3402/ejpt.v4i0.21305

**Published:** 2013-06-06

**Authors:** Stuart Turner

**Affiliations:** The Trauma Clinic, London, UK

**Keywords:** psychotraumatology, Europe, history, ESTSS, PTSD

## Abstract

This paper outlines a personal account of the growth of the field of traumatic stress in Europe, especially with the history of major disasters in the 1980s, the first European Conference in Lincoln in 1988, the formation of European Society for Traumatic Stress Studies and its subsequent development, for example, with a federal structure and its own journal, and most important of all the way that the field as a whole has matured.

My own role in this field probably began as an evening volunteer, in the mid-1980s, in an organization in London working with survivors of torture (then called the Medical Foundation for the Care of Victims of Torture, now renamed as Freedom from Torture). I held a university appointment at the time—undertaking early imaging research into schizophrenia, so this was very much an out of hours and a personal commitment. It was a time for rapid learning as this was a unique service in the UK, and I was the first psychiatrist to work there regularly. It felt like pioneering (and sometimes professionally lonely) work.

In November 1987, during an evening clinic at the Medical Foundation, I heard a lot of sirens outside, later learning that these were emergency services heading to Kings Cross railway station where a fire had broken out killing many people. Suddenly I found myself developing a programme of research and service provision for disaster survivors, working alongside my academic colleagues, Rachel Rosser and James Thompson (and later in collaboration with other European disaster services through the initiative of Wolfram Schüffel). The whole direction of my professional life had irrevocably changed.

The origins of European Society for Traumatic Stress Studies (ESTSS) can be traced back to Roderick Ørner and his meeting in Lincoln in 1988, which I attended (Ørner, 2013). With his usual wisdom and prescience, he called this the *first* European Conference on Traumatic Stress (ECOTS). Of course having had the first ECOTS, we were all keen that there should be more. Somehow I became involved in this process, working closely with colleagues who became good friends—especially Wolter de Loos, Atle Dyregrov and Roderick Ørner, with Lars Weisaeth sitting in the background as our elder statesman and mentor. We started out with the aim of producing a European Network that could continue to provide biennial meetings, and in time I became chair of what we called the European Trauma Foundation, in effect the precursor organization to ESTSS.

This was still a time when research focused on describing, in relatively simple terms, how people reacted to different types of experience and on what might be helpful to them. I recall the limitations of our knowledge at the time—as demonstrated in a review by Solomon et al. published in 1992, which found only 11 (small) randomized treatment trials. These authors concluded that drug studies showed a modest but clinically meaningful effect on posttraumatic stress disorder (PTSD). Prolonged exposure appeared to have stronger effects but caution was expressed about the risk of complications. As far as other psychological techniques were concerned, they could only conclude that these “may hold promise”. So this was a period of discovery—inevitably with an openness to many new ideas, but in the absence of a substantial amount of hard science.

Of course, we in Europe have a long tradition of interest in the traumatic neuroses, although the names we apply to these conditions have changed over time (e.g., Trimble, 1981). It was therefore no surprise that we should understand the importance of the concept of PTSD when introduced into DSM-III. Equally it was no surprise that Europeans should take a broader and sometimes more sceptical approach to diagnostic classification, aware of many of the complexities of trauma reactions, for example, with the legacy of trench warfare in the first world war or the serious issues faced by holocaust survivors, refugees and displaced people, and their families, within our culturally diverse continent. I recall many lively discussions in these early days—for example, concerning Antonovsky and his concept of salutogenesis, or Louis Crocq describing the history of the concept in France. By the time ESTSS was formed, Europe also had to confront the effects of the wars in the former Yugoslavia.

During this time, there were other developments. I had also become involved with the International Society for Traumatic Stress Studies (ISTSS). Charles Figley and John Wilson (first and second presidents of what was then the STSS) had attended the first ECOTS in Lincoln. I went to my first ISTSS meeting in San Francisco in 1989, when Yael Danieli was its (third) president. Then, in 1991, with James Thompson I established the Traumatic Stress Clinic, in London, initially under a national contract directly with the UK Department of Health. This was a direct response to research I had undertaken with Jeffrey Easton into the needs of the UK civilians held as part of the “human shield” following the Iraqi invasion of Kuwait. This clinic later became a model for others to use as services developed regionally across the UK, especially when we were joined by a pioneering children's trauma service led by Dora Black.

The birth of ESTSS was certainly not altogether straightforward and there were sometimes heated negotiations especially with colleagues in ISTSS but by 1993, Charles Marmar (as president of ISTSS) and I (as chair of the European Trauma Foundation) had achieved agreement on a way forward, which appeared to respect everyone's needs. This meant that at the third ECOTS in Bergen, we could announce the formation of a new European Society—the ESTSS.

Bergen was for many years my favourite conference on trauma (probably until my ISTSS presidential meeting in Chicago in 2008). The third ECOTS was small enough to achieve a real sense of intimacy and yet attracted most of the leading players in the field at the time. Atle Dyregrov was a wonderful host. Wolter de Loos had volunteered to take on the crucially important role of being the first president of ESTSS and to steer its early development. I also came to know Ueli Schnyder, who was persuaded to become ESTSS treasurer and who has become one of my closest friends and collaborators over the years both in ESTSS and later in ISTSS (Schnyder, 2013).

For me, 1993 was also an important year for other reasons. As well as helping to give birth to ESTSS, it was also the year when my first child was born. Like any child, ESTSS itself has had to grow and develop through its infancy and adolescence, sometimes kicking and screaming. I recall the year when so many of the founding board members had to stand down, having served out our maximum terms. It felt as if the society we had nurtured was standing on its own. Since then, through the effort and commitment of its presidents and boards, ESTSS has really flowered into its present form—a federation of trauma societies, many recreating much of the growth and enthusiasm of ESTSS in its early days. In particular, ESTSS now has its own journal—a long held ambition but not realized until Miranda Olff, its first editor, took the initiative (Olff, 2013). The *European Journal of Psychotraumatology* has already achieved inclusion in PubMed and PsychLit and has an unofficial impact factor in excess of 1.5.

Our field has also matured. Now, we have largely moved beyond answering simple questions about the nature and frequency of reactions to trauma (although perhaps there is still too much emphasis only on the negative sequelae) and we have begun to explore more detailed hypotheses concerning the neuropsychology and neurophysiology of traumatic stress reactions as well as improving and systematically evaluating our treatments. In my own work, I have moved from simply seeking to understand more about how torture survivors feel about their experiences. My current focus, with Jane Herlihy, who directs the Centre for the Study of Emotion and Law (csel.org.uk), is on gathering, through primary and secondary research, a body of general psychological knowledge to help officials achieve better and more just decisions when faced by an asylum seeker (e.g., Herlihy et al., 2012).

ESTSS is, and I hope will continue to be, a focus for rich and diverse discussions as well as a provider of peer support for all of us facing research, educational or (especially) clinical challenges in our work. I salute its twentieth anniversary.
